# *In vitro* anti-trypanosomal effects of selected phenolic acids on *Trypanosoma brucei*

**DOI:** 10.1371/journal.pone.0216078

**Published:** 2019-05-02

**Authors:** Cynthia Mmalebna Amisigo, Christine Achiaa Antwi, Jonathan Partt Adjimani, Theresa Manful Gwira

**Affiliations:** 1 West African Centre for Cell Biology of Infectious Pathogens, College of Basic and Applied Sciences, University of Ghana, Legon, Accra, Ghana; 2 Department of Biochemistry, Cell and Molecular Biology, College of Basic and Applied Sciences, University of Ghana, Legon, Accra, Ghana; Cleveland State University, UNITED STATES

## Abstract

African trypanosomiasis remains a lethal disease to both humans and livestock. The disease persists due to limited drug availability, toxicity and drug resistance, hence the need for a better understanding of the parasite’s biology and provision of alternative forms of therapy. In this study, the *in vitro* effects of phenolic acids were assessed for their trypanocidal activities against *Trypanosoma brucei brucei*. The effect of the phenolic acids on *Trypanosoma brucei brucei* was determined by the alamarBlue assay. The cell cycle effects were determined by flow cytometry and parasite morphological analysis was done by microscopy. Effect on cell proliferation was determined by growth kinetic analysis. Reverse Transcriptase quantitative Polymerase Chain Reaction was used to determine expression of iron dependent enzymes and iron distribution determined by atomic absorption spectroscopy. Gallic acid gave an IC_50_ of 14.2±1.5 μM. Deferoxamine, gallic acid and diminazene aceturate showed a dose dependent effect on the cell viability and the mitochondrion membrane integrity. Gallic acid, deferoxamine and diminazene aceturate caused loss of kinetoplast in 22%, 26% and 82% of trypanosomes respectively and less than 10% increase in the number of trypanosomes in S phase was observed. Gallic acid caused a 0.6 fold decrease, 50 fold increase and 7 fold increase in the expression levels of the transferrin receptor, ribonucleotide reductase and cyclin 2 genes respectively while treatment with deferoxamine and diminazene aceturate also showed differential expressions of the transferrin receptor, ribonucleotide reductase and cyclin 2 genes.

The data suggests that gallic acid possibly exerts its effect on *T*. *brucei* via iron chelation leading to structural and morphological changes and arrest of the cell cycle. These together provide information on the cell biology of the parasite under iron starved conditions and provide leads into alternative therapeutic approaches in the treatment of African trypanosomiasis.

## Introduction

African trypanosomiasis (AT) is an infectious disease that affects humans, domestic and wild animals in sub-Saharan Africa and it is transmitted by the tsetse fly [1]. *Trypanosoma brucei (T*. *brucei)* is responsible for causing AT in both cattle and humans [2]. The subspecies of *T*. *brucei* which includes *T*. *brucei gambiense* and *T*. *brucei rhodesiense* cause the chronic form of sleeping sickness in West and Central Africa and the acute form of the disease in East and Southern Africa respectively, with about 60 million people being at risk [3]. *Trypanosoma brucei brucei* is one of the causative agents of Animal African Trypanosomiasis (AAT) or nagana in cattle. About 55 million cattle are at risk with the disease leading to a loss of three million animals annually [4]. Due to the antigenic variation exhibited by the parasites, there is currently no vaccine against trypanosomes hence the mode of treatment is mainly by chemotherapy [5]. Drugs currently in use are toxic, have harmful side effects and are becoming less effective due to resistance. Hence the urgent need for the development of new anti-trypanosomal therapeutics which are safe and efficacious.

Phenolic acids are abundant plant secondary metabolites and there have been reports on their iron chelating properties [6, 7]. There are however only a few reports on their effects on the parasite’s biological activities. Trypanosomes require sufficient amount of intracellular iron for cellular activities such as DNA synthesis and energy metabolism. Studies have shown the trypanocidal activity of both synthetic and siderophore derived iron chelators. The iron chelator, deferoxamine, have been shown to inhibit the growth of parasites *in vitro*, affect the activity of ribonucleotide reductase [8, 9] as well as the G1-S phase of the cell cycle [10, 11]. The differential expression of the parasite’s transferrin receptor [12, 13] and cyclin genes [11] in response to iron deprivation has also been reported. Deferoxamine has however shown some level of toxicity against mammalian cell lines [8, 9]. In this study, six phenolic acids with iron binding potentials were investigated for their trypanocidal and cytotoxic effects in *T*. *brucei*. The phenolic acids are grouped into two classes: the hydroxybenzoic acids and the hydroxycinnamic acid and derivatives. In addition to the cell cycle analysis and measurement of the expression levels of the transferrin receptor, ribonucleotide reductase and the cyclin gene, we also investigated the effect of the chelators on the parasites’ morphology and the mitochondrial membrane integrity.

## Materials and methods

### Trypanosome strains and culture

Bloodstream forms of *T*. *brucei brucei* GUTat 3.1 cell lines were cultured in HMI-9 media [14] supplemented with 10% FBS, β-mercaptoethanol and streptomycin/penicillin. The cell cultures were grown at 37°C in a humidified atmosphere containing 5% CO_2._

### Test compounds

**Fig 1:**
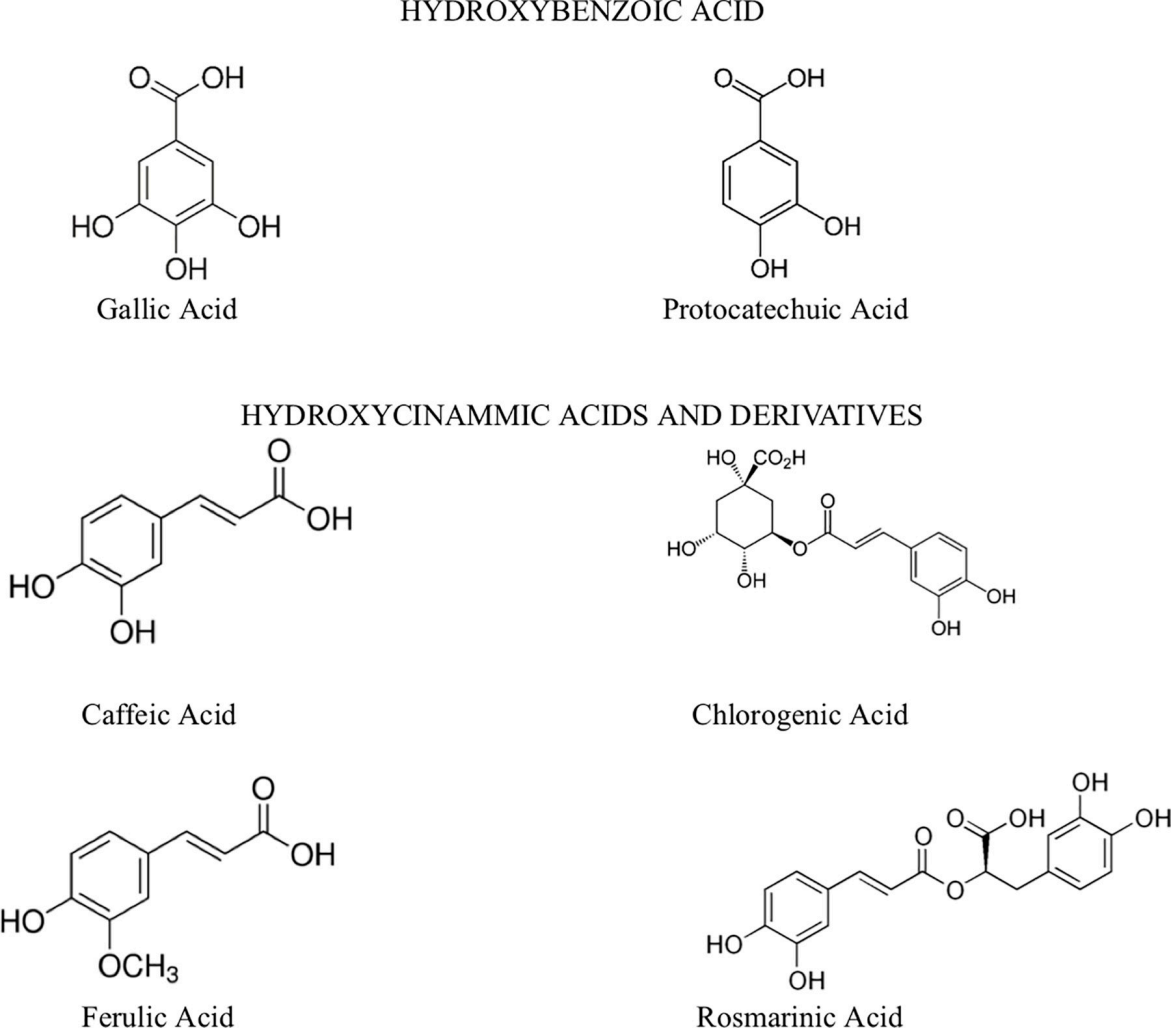
Structures of selected phenolic compounds.

All test compounds used, gallic acid (#SLBQ0358V), protocatechuic acid (#BCBR7275V), caffeic acid (#SLBL7069V), ferulic acid (#BCBQ6979V), rosmarinic acid (#BCBS0686V), chlorogenic acid (#SLBL9959V) (Fig 1), deferoxamine mesylate (#BCBT4388) and diminazene aceturate (#SLBN4612V) were obtained from Sigma-Aldrich. Diminazene aceturate (known drug for Animal African Trypanosomiasis) and deferoxamine (a known iron chelator) were used as positive controls. The compounds were selected based on their structures and iron binding affinities. Stock solutions of the compounds were prepared in dimethyl sulfoxide (DMSO) (Sigma-Aldrich) and working solutions in distilled water.

### Compound sensitivity test

Sensitivity test of the compounds against bloodstream forms *T*. *brucei brucei* was performed using the alamarBlue assay. Compounds were serially diluted in a flat bottom 96 well plate (Costar) with HMI-9 medium. Trypanosomes were cultured overnight to a density of 1x10^6^ cells/ml and a trypanosome cell suspension (100 μl) was added to the plates to give a final parasite density of 4000 parasites/ml. The plates were incubated for 72 hours at 37°C in 5% CO_2_. After 5 hours a final concentration of 44 μM of resazurin sodium salt (Sigma-Aldrich) in phosphate buffered saline was added to each well and the absorbance measured at 570 nm using the Varioskan Lux Elisa plate reader. Data was analyzed using Graphpad prism software (version 6). The IC_50_ values (concentration of compounds that inhibits 50% of growth) were determined. The IC_50_ reported for the compounds are the averages from three independent experiments. Gallic acid which showed a significant inhibitory effect on the parasites was selected for morphological studies, cell cycle and gene expression analysis.

### Analysis of parasite growth

Trypanosomes were seeded at an initial density of 1.0x10^5^ parasites/ml, counted using a haemocytometer (Sigma-Aldrich) and subcultured every 24 hours for a period of 5 days in the absence or presence of deferoxamine at IC_50_, 2X IC_50_ and 4X IC_50_, gallic acid at 1/4 IC_50_, ½ IC_50_ and IC_50_ and diminazene aceturate at ½ IC_50_ and IC_50_. A cumulative growth curve was plotted using Microsoft excel 2015.

### Morphological analysis

*T*. *brucei* cells (5x10^6^) treated with or without compounds (deferoxamine (2X IC_50_,), gallic (½ IC_50_) and diminazene aceturate (½ IC_50_)) were harvested by centrifuging at 2700 rpm for 8 minutes. The pelleted trypanosomes were washed with Voorhei’s modified PBS (vPBS) centrifuged and resuspended in 1 ml vPBS. One milliliter of 8% paraformaldehyde was added and incubated for 10 minutes. An additional 20 μl of 10% Triton X-100 (Sigma-Aldrich) was added to trypanosomes suspension and incubated further for 10 minutes. PBS (12 ml) was added and centrifuged at 2700 rpm for 8 minutes. The pellet was resuspended in 150 μl of PBS of which 50 μl was spread on polysine coated slides for 1 hour. The slides were washed twice for 5 minutes and 100 μl of 1 μg/ml DAPI (4, 6-Diamidino-2-phenylindole dihydrochloride) was added and incubated for 10 minutes. The slides were washed again in PBS for 5 minutes after which 10 μl of mounting media was added and covered. The nucleus and kinetoplast was examined with an Olympus Fluorescent microscope (100x magnification).

To study the effect of the compounds on the mitochondria, up to 2.5x10^5^ trypanosomes were cultured with different concentration of the compounds (deferoxamine at 2X IC_50_, 4X IC_50_, 9X IC_50_, gallic acid at ½ IC_50_, IC_50_ and 2X IC_50_ and diminazene aceturate at ½ IC_50_ and IC_50_) for 24 hours. The trypanosomes were harvested by centrifugation at 2700 rpm for 10 minutes and resuspended in 1ml serum free HMI-9/1% BSA. Ten microliters of MitoTracker Red CMXRos (Thermo fisher Scientific) was added to give a final concentration of 100 nM and incubated for 30 minutes. The trypanosomes were pelleted and resuspended in 1 ml serum free HMI-9/1% BSA and incubated for another 30 minutes. Pelleted parasites were suspended in a mixture of 0.5 ml vPBS and 0.5 ml 6% paraformaldehyde and incubated at 4°C for 1 hour. The trypanosomes were washed and resuspended in PBS and 50 μl was spread on a polysine coated slide, allowed to air dry and DAPI stain (1 ug/ml) was added. The slides were washed in PBS for 5 minutes, 10 μl of mounting media was added and the cover slip sealed with nail polish. The mitochondrion was examined using the Olympus Fluorescent microscope (100x magnification).

### Cell cycle analysis

Trypanosomes (1x10^5^/ml) were cultured for 24 hours with deferoxamine at 2X IC_50_, gallic acid at ½ IC_50_, diminazene aceturate at ½ IC_50_ and harvested by centrifugation at 2700 rpm, then washed twice with PBS and fixed in 70% cold ethanol at -20°C overnight. The trypanosomes were then washed twice in PBS and 200 μl of guava cell cycle reagent (Sigma-Aldrich) was added and incubated in the dark for 30 minutes. Cell cycle analysis was done using the LSRFortessa X-20 flow (BD Biosciences) and FlowJo v10 software. At least 5000 trypanosomes were counted for each measurement.

### Gene expression analysis using RT q-PCR

Treated and untreated parasites were harvested, and total RNA extracted from 5x10^7^ trypanosomes using the ZymoQuick-RNA MiniPrep Plus (Zymo Research, USA) following the manufacturer’s protocol after an overnight incubation with TRIzol reagent at -80°C. RT-qPCR was carried out using the Luna Universal One-Step RT-qPCR kit (New England Biolabs) following the manufacturer’s instructions. The final concentration of the reaction mixture included: 1X of Luna Universal One-step reaction mix, 0.4 μM of forward and reverse primers and 1X of Luna Warmstart RT Enzyme Mix and 1 μg of template RNA. Data generated was analyzed using the Quant Studio 3 and 5 Real-Time PCR Systems (Thermo Fisher Scientific) and the results represent the average of two independent experiments. Primers used targeted three genes involved in iron metabolism (cyclin 2 gene, ribonucleotide reductase and transferrin receptor). Histone H2A was used as the endogenous control. The primer sequences are shown in Table 1.

**Table 1:** Sequences of primers used of RT-qPCR.

Gene ID	Gene name	Sequences
Tb927.7.2820	Histone H2A putative (FW)	AGTGAAGAAGGCATCGAAGG
	Histone H2A putative (RV)	CACGGATAGCTCCAGCAGTT
Tb927.9.15680	Transferrin receptor subunit (FW)	GATCGTGGGTGTTGACCTCT
	Transferrin receptor subunit (RV)	CAGATATGTTTGCGGGGACT
Tb927.11.14080	Cyclin 2 (FW)	GTGCTCACCAGAATGCTTCA
	Cyclin 2 (RV)	GCCACCAATACCTGCAAAGT
Tb927.11.12790	Ribonucleoside-diphosphate reductase small chain (FW)	CGTCATTGCAACTCGAAGAA
	Ribonucleoside-diphosphate reductase small chain (RV)	CTGCGTCCAGAGAGAAAACC

All sequences are in the 5’to 3’ direction

FW = Forward primer

RV = Reverser primer

### Iron content analysis

Trypanosomes were treated with deferoxamine at 2X IC_50_ and gallic acid at ½ IC_50_ for 24 hours and the iron content of the parasites estimated as described by [15]. Briefly, 5x10^6^ trypanosome cells were harvested and suspended in 100 μl of sterile water and 10 μl was used for protein content determination as described previously [16]. Hundred microliters of spent media (media collected after harvesting the trypanosomes) was analyzed for iron. Both the media and trypanosomes were treated with 200 μl of 100% nitric acid at 80°C for 1 hour and incubated at 20°C overnight to digest. After digestion, 60 μl of 30% hydrogen peroxide was added to stop the reaction. Sterile water was added to give a final volume of 2 ml. The iron content was measured by atomic absorption spectroscopy (Analyst 300; PerkinElmer, Foster City, CA, USA).

### Cytotoxicity of compounds against mammalian cells

Toxicity of compounds to Raw 264.7 macrophage cells was evaluated. Briefly, the macrophages were seeded into 96-well plates at ~1x10^5^ macrophages/ml in DMEM supplemented with 10% FBS, 100X penicillin/streptomycin, 2g/l NaHCO_3_ and incubated at 37°C for 24 hours in 5% CO_2_. The Raw 264.7 macrophage cells were treated with varying concentrations of deferoxamine, gallic acid and diminazene aceturate (0–50 μg/ml) for 48 hours at 37°C in 5% CO_2_. Cell viability was estimated by MTT reagent and absorbance was measured at 570 nm using the Varioskan Lux plate reader. Cytotoxicity (CC_50_) values were estimated using the Graphpad prism 6 software and the selectivity index (SI) was calculated as the ratio of the CC_50_ to IC_50_ values.

## Results

### *In vitro* trypanocidal activity of compounds against *Trypanosoma brucei*

To investigate the effects of the compounds on cell viability, trypanosome cells were incubated with varying concentrations of compounds for 72 hours. A total of six compounds (caffeic acid, chlorogenic acid, ferulic acid, gallic acid, protocatechuic acid and rosmarinic acid) were used. Deferoxamine and the diminazene aceturate were used as positive controls. The IC_50_ values for each iron chelator and control compounds were determined. In this study, gallic acid and rosmarinic acid exhibited moderate anti-trypanosomal activity compared to deferoxamine and diminazene aceturate. The IC_50_ obtained for diminazene aceturate, deferoxamine and gallic acid were 0.1 μM±0.02, 3.3 μM±0.26 and 14.2 μM+1.5 respectively (Table 1). Caffeic acid, chlorogenic acid, ferulic acid and protocatechuic acid showed negligible anti-trypanosomal activity with IC_50_ >100 μM (Table 2).

**Table 2:** Anti-trypanosomal activity of compounds on *Trypanosoma brucei brucei*.

Test compounds	IC_50_(μM)±SD
Diminazene Aceturate (DA)^a^	0.1 ± 0.02
Deferoxamine (DFO)^b^	3.3 ± 0.26
Gallic Acid (GA)	14.2 ± 1.5
Rosmarinic Acid	17.31± 0.08
Caffeic Acid	>100
Ferulic Acid	>100
Chlorogenic Acid	>100

^a^ Standard anti-trypanosomal drug

^b^ Standard iron chelator

### Effect of compounds on the proliferation of *Trypanosoma brucei*

**Fig 2:**
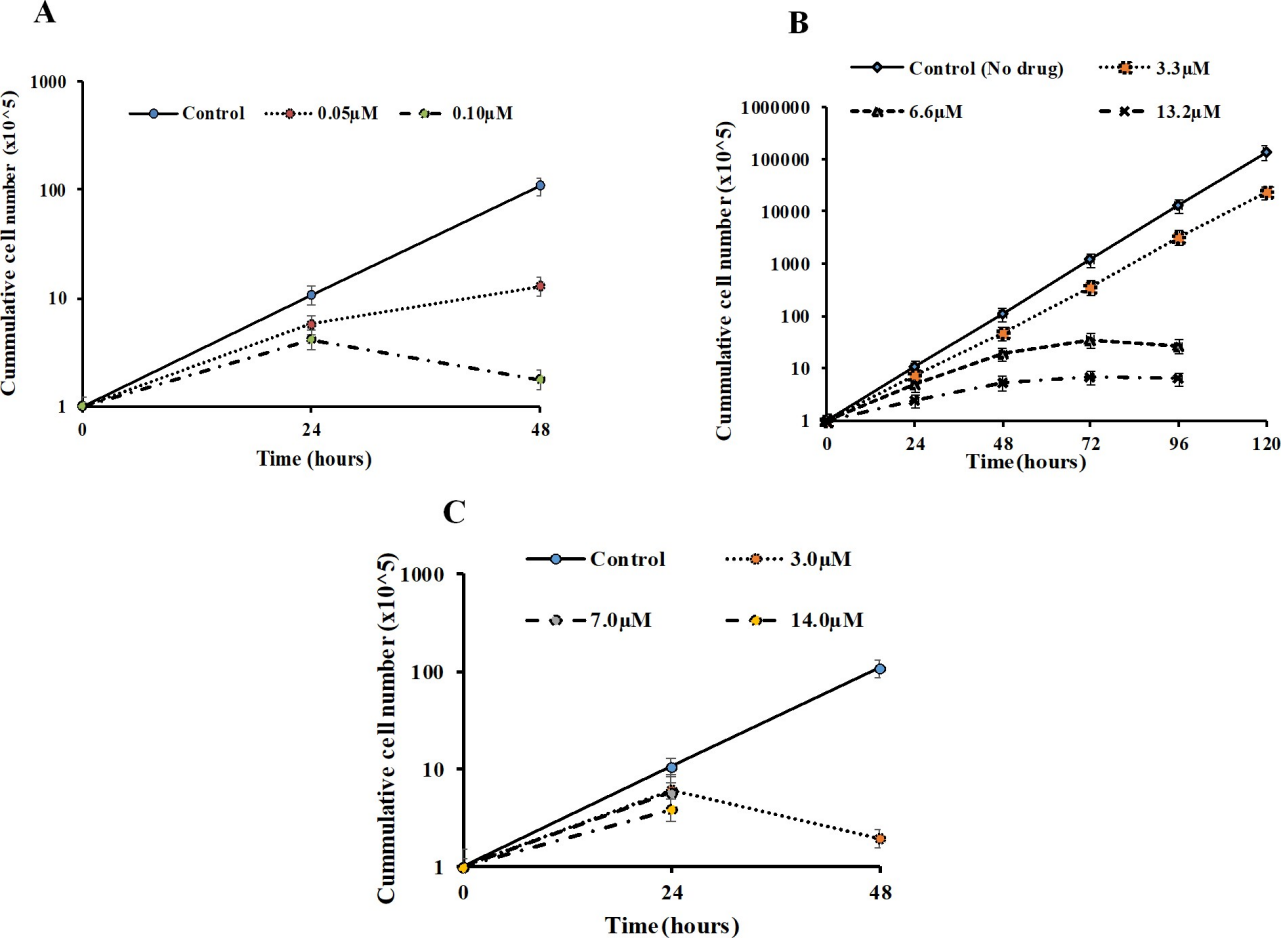
Dose dependent effect of compounds on the proliferation of *Trypanosoma brucei brucei*. Cumulative growth analysis of trypanosomes in HMI-9 media and 10% FBS in the absence or presence of different concentrations of compounds. **(A)** Diminazene aceturate **(B)** Deferoxamine **(C)** Gallic acid.

To determine the effect of the active compounds on the growth rate of the parasite, trypanosomes were cultured in the absence or presence of different concentrations of the compounds (diminazene aceturate, 0.05 μM and 0.1 μM; deferoxamine, 3.3 μM, 6.6 μM, and 13.2 μM; gallic acid, 3 μM, 7 μM, 14 μM), and the cell density measured by counting daily for 5 days (Fig 2). Treatment with increasing concentrations of the compounds resulted in reduction in cell proliferation (Fig 2). An exponential growth was observed for untreated cells with trypanosomes dividing between 8–12 times in 24 hours. However, there was reduction in cell density when trypanosomes were treated with diminazene aceturate and gallic acid for 24 hours at the different concentrations (Fig 2A and 2C). A more drastic reduction in cell density was observed at 48 hours of treatment with diminazene aceturate and gallic acid. There was complete cell death when trypanosomes were treated with gallic acid at the IC_50_ concentration (Fig 2C). There was reduction in cell proliferation when treated with 2X IC_50_ and 4X IC_50_ concentrations of deferoxamine, but treatment with IC_50_ concentrations did not have any significant effect on the cell proliferation (Fig 2B).

### Effect of compounds on the cell morphology, DNA synthesis and mitochondria integrity

**Fig 3:**
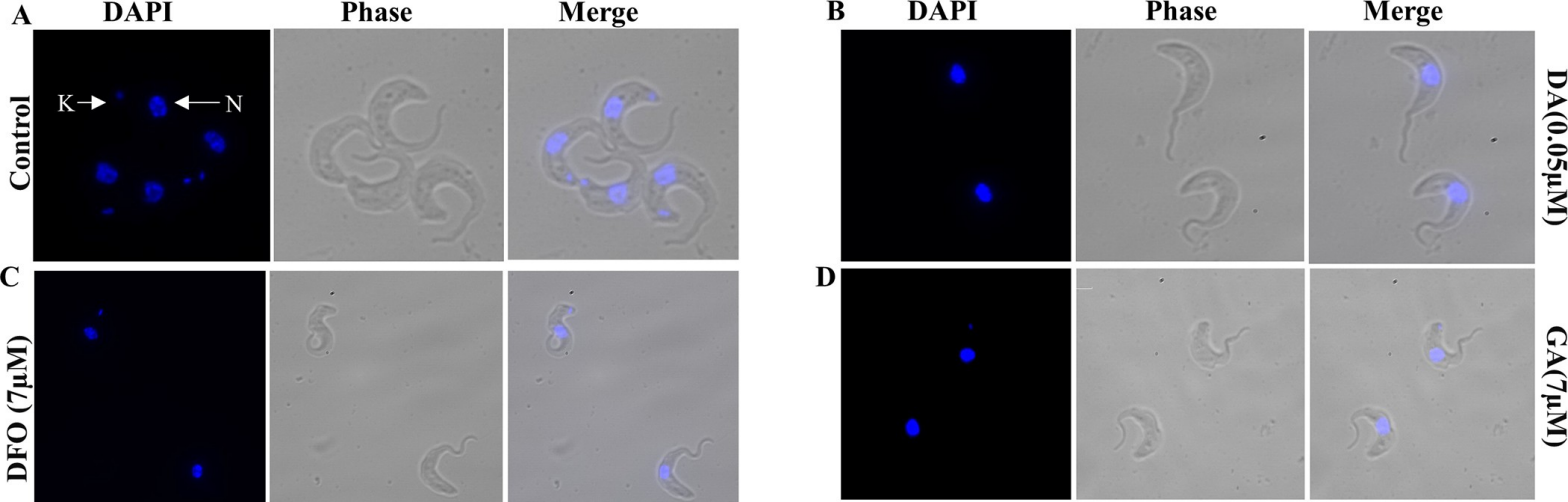
Effects of compounds on trypanosome morphology and DNA synthesis. *T*. *b*. *brucei* cells were treated with ½ IC_50_ diminazene aceturate (DA); 2X IC_50_ deferoxamine (DFO) and ½ IC_50_ gallic acid (GA) for 24 hours, stained with DAPI for DNA (blue) and cell morphology visualized using the fluorescent microscope (100x). The nucleus is indicated as N and the kinetoplast as K.

To assess the effect of the selected compounds on parasite morphology and synthesis of DNA by the parasite, trypanosomes were cultured in the absence or presence of different concentrations of compounds for 24 hours, stained with DAPI, and observed under the fluorescent microscope. Most of the untreated trypanosomes had long slender morphology with intact flagella and had at least a nucleus and a kinetoplast (Fig 3A). However, when treated with ½ IC_50_ concentration of diminazene aceturate, 82% of trypanosome cells lost their kinetoplast (1NK0) (Fig 3B) while treatment with 2X IC_50_ of deferoxamine and ½ IC_50_ of gallic acid resulted in 26% and 22% of trypanosomes respectively losing their kinetoplast (Fig 3C and 3D).

**Fig 4:**
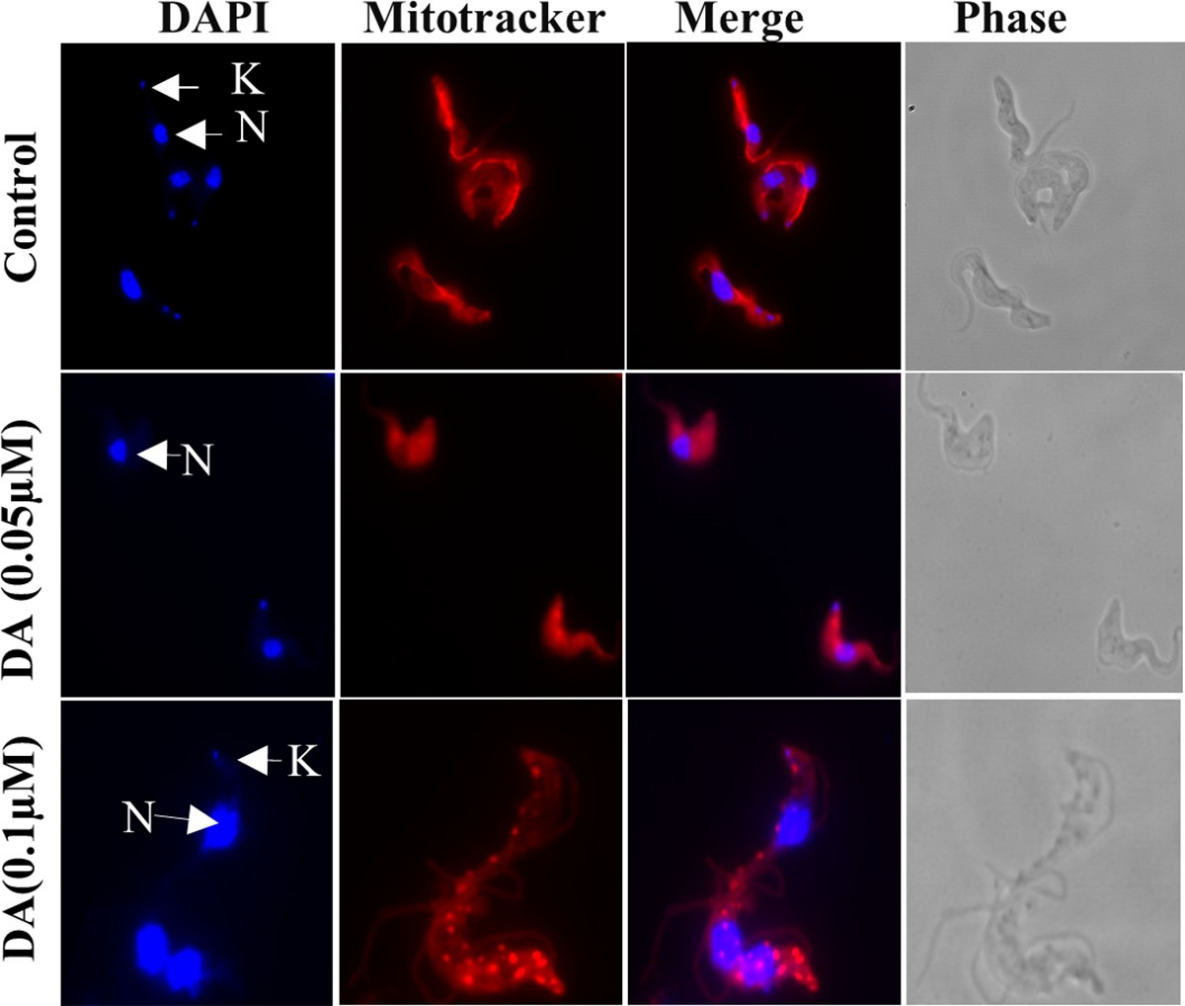
Effect of diminazene aceturate on the mitochondrial membrane integrity and *T*. *b*. *brucei* morphology. Trypanosomes were treated with diminazene aceturate (DA) [0.05μM; 0.1μM], mitochondria labelled with Mitotracker red dye (red), counterstained with DAPI to detect DNA (blue), and cell morphology visualized using the fluorescent microscope (100x). The concentrations used were ½ IC_50_, IC_50_.

**Fig 5:**
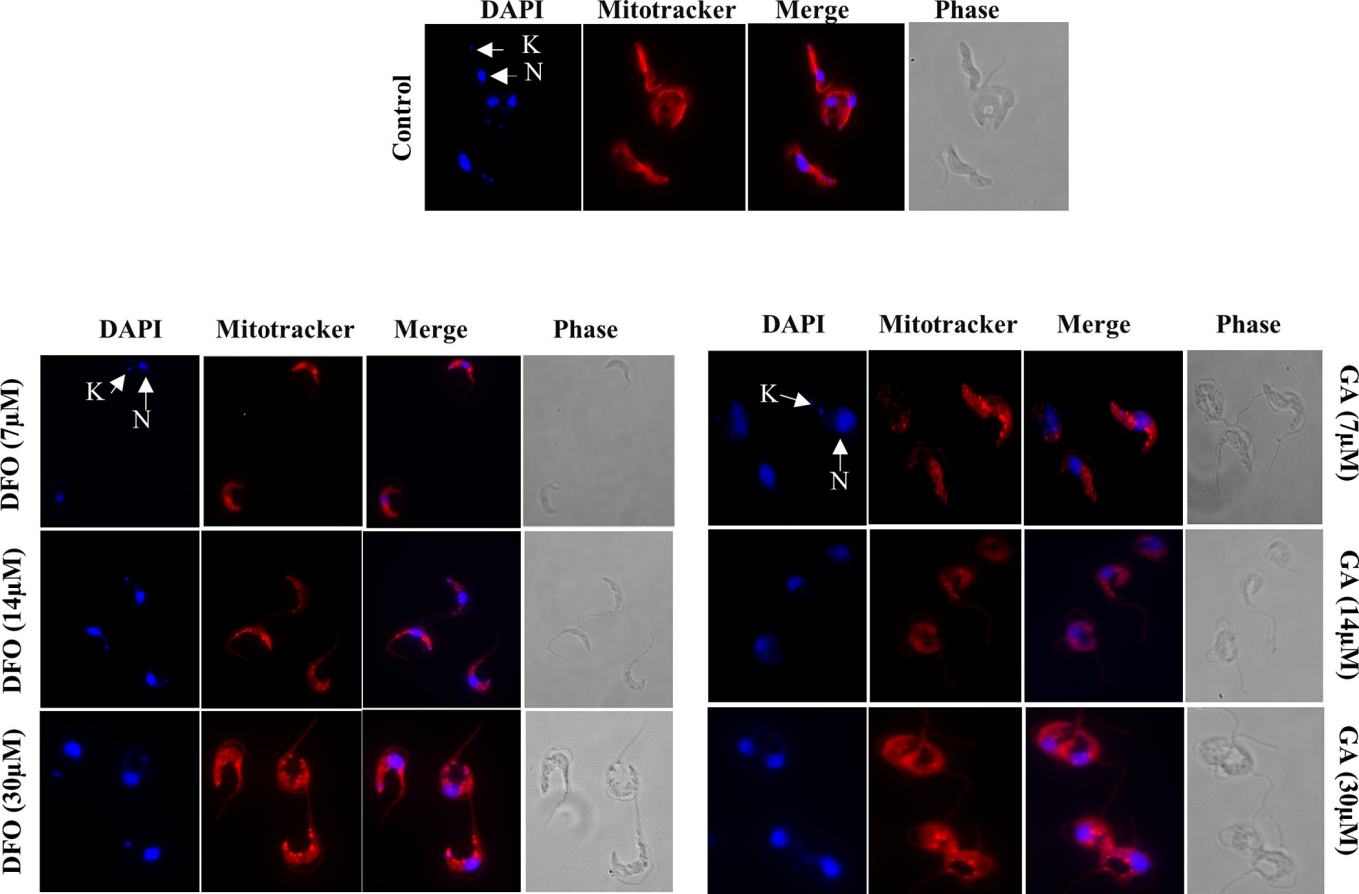
Effect of deferoxamine and gallic acid on the mitochondrial membrane integrity and *T*. *b*. *brucei* morphology. Trypanosomes were treated with deferoxamine (DFO and GA) [7μM; 14μM; 30 μM] labeled for mitochondria with Mitotracker red dye (red), counterstained with DAPI for DNA (blue) and cell morphology visualized using the fluorescent microscope (100x). The concentrations used were 2x IC_50_, 4x IC_50_, 9x IC_50._ and ½ IC_50_, IC_50_ and 4x IC_50_ for DFO and GA respectively. The control samples used here are the same as those in Fig 4 (All the microscopy experiments were done in parallel).

The effect of compounds on mitochondrial membrane integrity was assessed by growing trypanosomes in the presence or absence of compounds for 24 hours, stained with the MitoTracker dye and trypanosomes analysed under the fluorescence microscope. The dye makes use of negative membrane potential by binding to the thiol groups in the mitochondria. Trypanosomes with an intact membrane potential are able to retain the dye while those that have defect in the mitochondria membrane will not fully retain the dye leading to leaking of dye into the cytoplasm observed as red spots in the cytoplasm. The untreated controls had their mitochondrion stained with the MitoTracker dye which revealed the mitochondrion that run from the anterior to the posterior end of the cell. The test compounds showed dose dependent changes in the mitochondrial membrane integrity as well as its morphology (Figs 4 and 5). About 90% of diminazene aceturate treated trypanosomes had a defect in the mitochondrial membrane resulting in the accumulation of the bright red aggregation of MitoTracker dye in the cytoplasm (Fig 4). Increasing concentration of deferoxamine (7.0 μM, 14.0 μM and 30.0 μM) resulted in the aggregation of dye in the cytoplasm of 70%, 97% and 98% of trypanosomes respectively (Fig 5). Trypanosomes treated with gallic acid at the respective concentrations (7.0 μM, 14.0 μM and 30.0 μM) showed 83%, 86% and 87% respectively of parasites having an accumulation of bright red aggregation of the dye in the cytoplasm (Fig 5). All treated trypanosomes had an altered or abnormal morphology which includes the rounding up of cells, detached cell body and an elongated flagellum (Figs 4 and 5).

### Cell cycle effects of treatment with compounds

**Fig 6:**
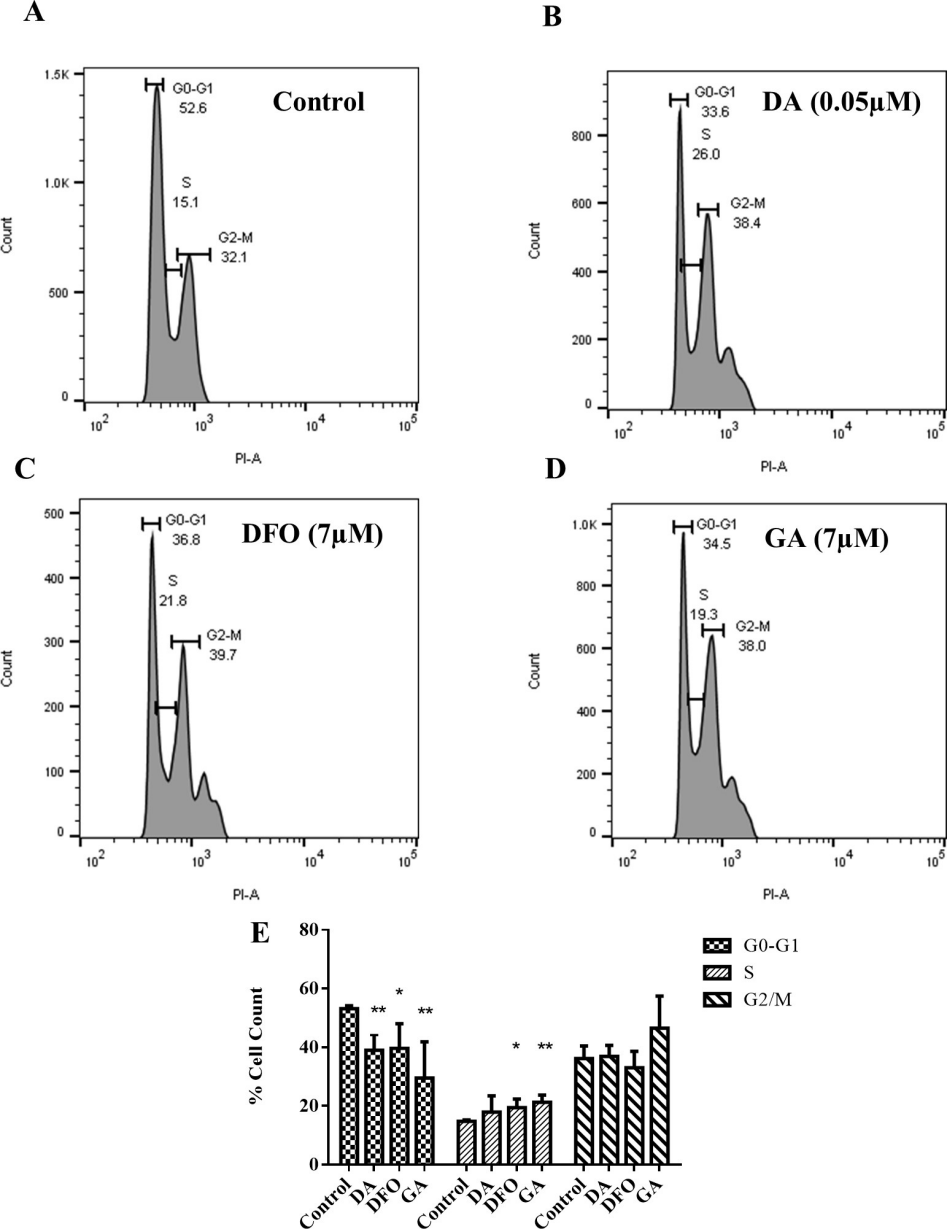
Effects of compounds on *T*. *brucei* cell cycle. Flow cytometry outputs **(A)** untreated control **(B)** diminazene aceturate (0.05 μM) **(C)** deferoxamine (7 μM) **(D)** gallic acid (7 μM). **(E)** Percentage cell count at each cell cycle phase. Results are represented as average ±SD. Statistical significance was determined using unpaired t test by comparing the treated and the control for each phase, p<0.05. Data in (E) represents an average of four independent experiments.

The effect of the compounds as well as the standard anti-trypanosomal drug on the cell cycle phases of *T*. *b*. *brucei* was evaluated. Trypanosomes were treated with different concentrations of compounds (diminazene aceturate, 0.05μM; deferoxamine, 7 μM; gallic acid, 7 μM) for 24 hours and analysed by flow cytometry (Fig 6A–6D). The untreated trypanosomes showed 53.2±0.98, 14.8±0.45 and 36.2±4.29% of cells at the G0-G1, S and G2-M phases respectively. The treated trypanosomes had lower percentage cell count at the G0-G1 phase (diminazene aceturate = 39.0±5.42, p value = 0.0024; deferoxamine = 39.6 ±8.38%, p-value = 0.0201; and gallic acid = 29.5±12.34%, p-value = 0.0092). There was no significant change in the percentage cell count in the G2-M phase for all compounds tested (Fig 6E). However, deferoxamine and gallic acid treated trypanosomes showed increase in the percentage cell count at S phase relative to the control (deferoxamine = 19.5 ±2.89%, p-value = 0.0182 and gallic acid = 21.3±2.44%, p-value = 0.0020).

### Treatment with compounds leads to differential expression of iron metabolic genes

**Fig 7:**
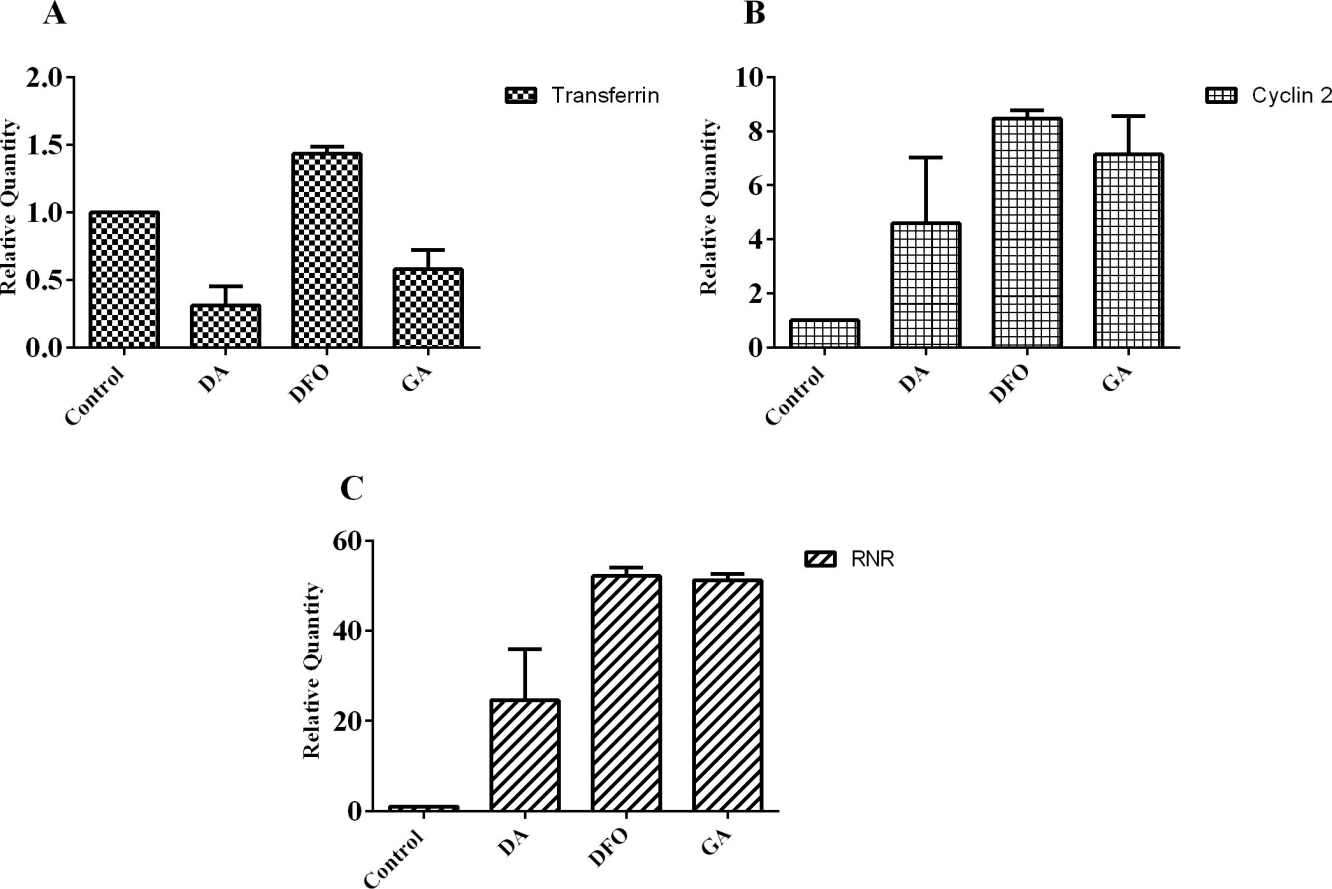
Relative gene expression of iron dependent genes. RT-qPCR analysis of mRNA expression of **(A)** Transferrin receptor **(B)** Cyclin 2 **(C)** Ribonucleotide reductase after treatment with diminazene aceturate (DA), deferoxamine (DFO) and gallic acid (GA) for 24 hours. The values are expressed as the relative quantity with respect to the control. The data represent the average of two independent experiments.

Reverse Transcriptase -qPCR was performed to compare the relative gene expressions of selected iron dependent genes in trypanosomes when treated with diminazene aceturate (0.05 μM), deferoxamine (7μM) and gallic acid (7 μM) for 24 hours (Fig 7). The change in mRNA expression level was determined by comparing to the untreated control. Varying degree of expressions of the iron dependent proteins in the presence of the compounds was observed. All treated trypanosomes showed an increase in the expression of the cyclin 2 (diminazene aceturate, 5 fold; deferoxamine, 8 fold and gallic acid, 7 fold) and ribonucleotide reductase gene (diminazene aceturate, 25 fold; deferoxamine, 52 fold and gallic acid, 50 fold) (Fig 7B and 7C). Interestingly we observed only a 1.4 fold increase in the transferrin receptor mRNA in the presence of deferoxamine and 0.3 fold and a 0.6 fold decrease in the diminazene aceturate and gallic acid treated trypanosomes respectively (Fig 7A).

### Effect of deferoxamine and gallic acid on the intracellular iron content of parasites

**Fig 8:**
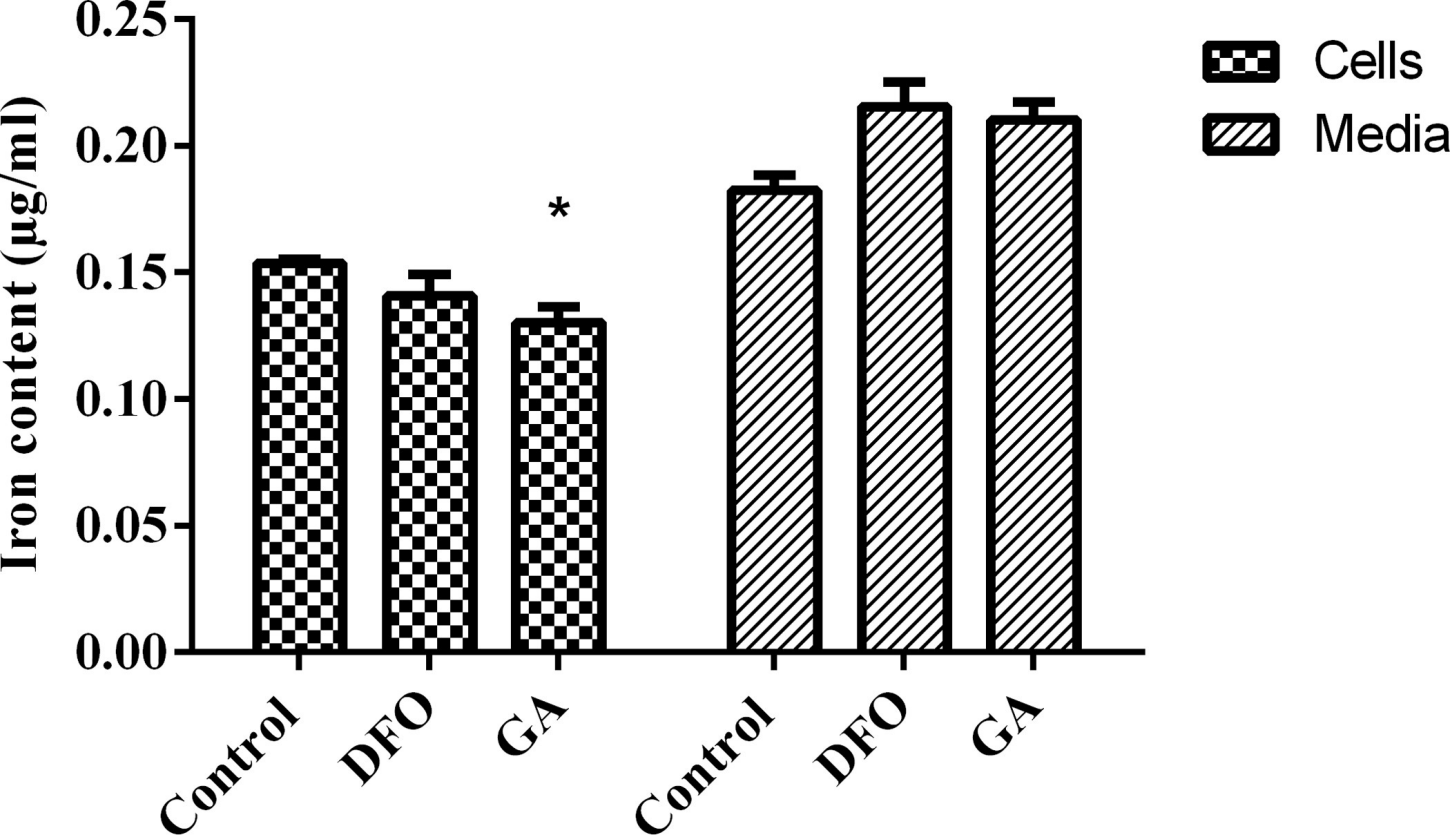
Effect of compounds on intracellular and extracellular iron content. Trypanosomes were treated with 7μM of deferoxamine (DFO) and gallic acid (GA). Significance was determined using the unpaired t test comparing treated and control, *p < 0*.*05*.

In order to assess whether the chelators were affecting the availability of iron to the parasites, the intracellular iron content (amount of iron within the trypanosomes) and the extracellular iron content (amount of iron in media) were determined by culturing trypanosomes in the presence of deferoxamine (7μM) and gallic acid (7 μM) for 24 hours and the iron content measured using the atomic absorption spectrometry. There was a reduction in the intracellular iron content in deferoxamine treated trypanosomes. Gallic acid also significantly reduced the intracellular iron content (deferoxamine = 0.1377±0.0042, p-value = 0.1695 and gallic acid = 0.1302±0.0061, p-value = 0.0354) (Fig 8).

### Cytotoxicity of compounds against mammalian macrophages

To determine the toxicity of the compounds against mammalian cells, macrophages were incubated with (0–50μg/ml) concentrations of diminazene aceturate, deferoxamine and gallic acid, and the cell viability determined by the MTT assay. The CC_50_ values generated were used to calculate the selectivity index (SI). Compounds with selectivity index greater than 10 are considered less toxic to the macrophages. All the test compounds were found to be toxic to the mammalian cells with selectivity index less than 10 (Table 3). However, the iron chelators were moderately toxic to the macrophages compared to diminazene aceturate, the currently used drug for treating Animal trypanosomiasis.

**Table 3:** Selectivity index (SI) of test compounds against macrophage cell lines.

Test Compounds	IC_50_(μM)±SD	CC_50_(μM)±SD	SI = CC_50_/IC_50_
Deferoxamine	3.3 ± 0.26	26.7±0.32	8.1
Gallic acid	14.2 ± 1.50	36.1±3.33	2.5
Diminazene aceturate	0.1 ± 0.02	0.0014±0.05	0.014

A selectivity index < 10 indicates toxicity to the macrophage relative to the parasites

## Discussion

Phenolic acids are a class of polyphenols that have been shown to have anti-protozoan, anti-bacterial and anti-cancer properties [17, 18] but only few studies have been done to evaluate their effect on the cell biology of trypanosomes. Of the phenolic acids used in this study, gallic acid showed the highest trypanocidal activity with IC_50_ comparable to what was obtained by Koide and colleagues [19]. Gallic acid significantly inhibited parasite growth whereas protocatechuic acid, also a hydroxybenzoic acid, did not show any significant activity against the trypanosomes. Factors that can affect the iron chelating properties of these phenolic compounds include the number of the hydroxy groups and the position of the hydroxyl groups on the aromatic ring [6]. The trypanocidal activity of gallic acid could be due to the presence of the extra hydroxyl group and its ability to form reactive oxygen intermediates in the parasite [19]. Although gallic acid showed trypanocidal activity in this study, interestingly, in other studies, gallic acid was inactive against other kinetoplastids such as *Leishmania donovani* and *Trypanosoma cruzi* [19, 20] and this could be due to the intracellular nature of these parasites. The amastigote forms of *Leishmania* and the amastigotes of *Trypanosoma cruzi* exists intracellularly in mammalian cells. Thus, to effectively kill the intracellular parasites, the iron chelators must be lipophilic to be able to transverse the host cells.

The iron binding abilities and antioxidant activities of these phenolic acids have been linked to the number and positions of the hydroxyl groups attached to the benzene ring [6]. The differences in trypanocidal activities observed between gallic acid and protocatechuic acid could be linked to the galloyl group of gallic acid. Hydroxybenzoic acids with galloyl group are better iron chelators and have shown stronger trypanocidal activity than those with the catechol groups as found in protocatechuic acid [6, 9]. All the hydroxycinnamic acid derivatives used in this study were either moderately active or inactive against the trypanosome which could be attributed to a mechanism of action other than iron chelation. The IC_50_ value obtained in this study for deferoxamine treated trypanosomes was similar to results obtained by other researchers [9, 21].

Apart from growth inhibition, the compounds appeared to affect the morphology of trypanosomes by altering the shape of the cell and organelles such as the kinetoplast and mitochondrion. The loss of kinetoplast in some of the treated trypanosomes did not appear to inhibit proliferation under normal culture conditions as observed for diminazene aceturate [22]. Diminazene aceturate acts by binding to the minor groove of the DNA, preventing replication [23] leading to growth inhibition and loss of the kinetoplast [24]. The effects observed with the treatment with the iron chelators could be attributed to activity of ribonucleotide reductase (an iron dependent enzyme involved in DNA synthesis). Iron chelation does not directly affect this enzyme but could prevent the association of iron to new ribonucleotide reductase apoproteins [8]. Although the loss of kinetoplast in the gallic acid treated trypanosomes was moderate there may be other biological pathways that may be affected by depletion of iron by gallic acid.

The observed accumulation of MitoTracker in the cytoplasm of the trypanosomes suggest a compromise of the integrity of the mitochondrion membrane. A fully functional mitochondrion will have an intact membrane potential leading to the internalization and the retention of the dye in the mitochondrion. In a defective or leaky mitochondrion, the membrane potential will be lost, and the dye will aggregate in the cytoplasm [20]. The generation of reactive oxygen species causes damage to the mitochondrion leading to a loss in its membrane potential [25]. Since the machinery for mopping up free radical is iron dependent, the deprivation of iron could cause a dysfunctional iron superoxide dismutase (Fe-SOD) hence parasites with this dysfunctional enzyme will be overwhelmed by these ROS and this can ultimately affect the mitochondrial membrane integrity. Some esters of gallic acid have also been shown to affect the mitochondrial membrane of *T*. *cruzi* [20]. A similar observation was made for diminazene aceturate treated trypanosomes but how this happens is unclear. However, pentamidine (an anti-trypanosomal drug) which is structurally similar to diminazene aceturate has been shown to act by affecting mitochondrial membrane. Pentamidine breaks down the membrane leading to a dysregulation in the intra and extra cellular calcium ion content of the cell [20] hence diminazene aceturate could be affecting the membrane potential of the mitochondrial membrane via the same mechanism.

The general cell proliferation process and the production of normal daughter cells involves a smooth progression of the cell cycle and this process is a combined effect of the cyclins, cyclin dependent kinases and their inhibitors [26]. The effects of iron chelators on the cell cycle have been shown to result from differential expressions of genes and their respective protein expressions. Iron chelators are known to cause cell cycle arrest in the S phase [10, 11, 27]. The relative increase in the cyclin 2 gene found in this study could account for the accumulation of trypanosomes in the S phase, emphasizing the importance of this gene in the G1/S phase transition. Upon treatment with the iron chelators deferoxamine and aroylhydrazone [11], observed an increase in the mRNA levels of the cyclin dependent inhibitor (p21), which plays an important role in G1 cell cycle arrest, there was however a decrease in its protein levels. This observation is surprising since in other iron depletion experiments, the levels of transcription correlated with the levels of protein expression [28].

The effects of the chelators observed in this study confirms the fact that adequate amount of intracellular iron is necessary for essential metabolic processes in the parasite [29]. Therefore, iron chelators which are able to selectively reduce the intracellular iron content of the parasite could be very promising drug candidates. From this study both deferoxamine and gallic acid reduced intracellular iron content of the parasites. The results obtained for deferoxamine also agree with data that suggested that deferoxamine acts by reducing intracellular iron content [8]. Since the gallic acid also showed effects similar to those observed for deferoxamine, it is most likely that it also functions as an intracellular iron chelator or inhibits parasites by other mechanism(s) not investigated in this study. Most pathogens have developed different ways of obtaining iron in an iron limited environment. For example, under iron starved conditions, *Leishmania amazonensis* upregulates its iron transporter (LIT-1) within the phagolysosome to increase its intracellular iron content [30]. The moderate upregulation of the transferrin receptor in the deferoxamine treated trypanosomes could be an indication of a compensation mechanism employed by the cells under iron starved conditions in order to increase their iron intake. The 1.4-fold increase in the expression of this receptor is comparatively lower than the 3-fold upregulation of the receptor in deferoxamine treated cells [12], which could be the result of the differences in the cell lines and the concentration of compound used. Studies by Mussmann and colleagues also reported a 5-fold increase in the transferrin receptor in response to transferrin starvation [13]. The expression of the trypanosome transferrin receptor is via the regulation of the ESAG 6 transcript at the post transcriptional level. In the absence of iron, the expression levels of ESAG 6 increases to about 3-fold and this causes a relative increase in the transferrin receptor [12] but the mechanism of the regulation still remains unclear. In this study however, a low expression of the transferrin receptor in gallic acid treated cells was observed even though it caused reduced intracellular iron content. This suggest that its trypanocidal activity might involve other mechanisms in addition to its iron chelation properties.

In the search for effective therapeutic agents, one requirement of a potential drug candidate is its relative toxicity against the host cells. An ideal drug must be selectively toxic to the parasites. However, in the absence of a better alternative, some drugs are still administered despite their toxicity. For example, diminazene aceturate is still used as an anti-trypanosomal despite its side effects and toxicity [24]. Similar to what was found in this study, iron chelators are generally more toxic to trypanosomes compared to mammalian cells *in vitro* [9, 19, 21].

## Conclusions

We have shown that phenolic acids with iron chelating properties exhibited good trypanocidal activities with moderate toxicity to mammalian cell lines *in vitro* compared to the currently used standard animal trypanosomiasis drug. Our data suggests a mechanism of action of deferoxamine, gallic acid and diminazene aceturate to potently inhibit growth of *Trypanosoma brucei* via perturbation of cell morphology and mitochondrion membrane integrity, and cell cycle arrest while increasing the expression of ribonucleotide reductase and cyclin 2 genes with differential expression of the transferrin receptor. Our data also provides information on the cell biology of *T*. *b*. *brucei* under iron deprivation and provides leads for alternative chemotherapeutic in the treatment of African trypanosomiasis.

## References

[1] Caljon G, Van Den Abbeele J, Stijlemans B, Coosemans M, De Baetselier P, Magez S. Tsetse fly saliva accelerates the onset of *Trypanosoma brucei* infection in a mouse model associated with a reduced host inflammatory response. Infect Immun. 2006;74(11):6324–30. 10.1128/IAI.01046-06 16954393 PMC1695494

[2] Basu S, Horakova E, Lukes J. Iron-associated biology of *Trypanosoma brucei*. Biochim Biophys Acta. 2016;1860(2):363–70. 10.1016/j.bbagen.2015.10.027 26523873

[3] Brun R, Blum J, Chappuis F, Burri C. Human African trypanosomiasis. Lancet. 2010;375(9709):148–59. 10.1016/S0140-6736(09)60829-1 19833383

[4] Tihon E, Imamura H, Van den Broeck F, Vermeiren L, Dujardin JC, Van Den Abbeele J. Genomic analysis of Isometamidium Chloride resistance in *Trypanosoma congolense*. Int J Parasitol Drugs Drug Resist. 2017;7(3):350–61. 10.1016/j.ijpddr.2017.10.002 29032180 PMC5645165

[5] Black SJ, Mansfield JM. Prospects for vaccination against pathogenic African trypanosomes. Parasite Immunol. 2016;38(12):735–43. 10.1111/pim.12387 27636100

[6] Andjelkovic M, Van Camp J, De Meulenaer B, Depaemelaere G, Socaciu C, Verloo M, et al Iron-chelation properties of phenolic acids bearing catechol and galloyl groups. Food Chemistry. 2006;98:23–31.

[7] Genaro-Mattos TC, Mauricio AQ, Rettori D, Alonso A, Hermes-Lima M. Correction: Antioxidant Activity of Caffeic Acid against Iron-Induced Free Radical Generation—A Chemical Approach. PLoS One. 2015;10(11):e0142402 10.1371/journal.pone.0142402 26098639 PMC4476807

[8] Breidbach T, Scory S, Krauth-Siegel RL, Steverding D. Growth inhibition of bloodstream forms of *Trypanosoma brucei* by the iron chelator deferoxamine. Int J Parasitol. 2002;32(4):473–9. 11849643 10.1016/s0020-7519(01)00310-1

[9] Merschjohann K, Steverding D. In vitro growth inhibition of bloodstream forms of *Trypanosoma brucei* and *Trypanosoma congolense* by iron chelators. Kinetoplastid Biol Dis. 2006;5:3 10.1186/1475-9292-5-3 16914038 PMC1563472

[10] Kicic A, Chua AC, Baker E. The desferrithiocin (DFT) class of iron chelators: potential as antineoplastic agents. Anticancer Drug Des. 2001;16(4–5):195–207. 12049478

[11] Le NT, Richardson DR. The role of iron in cell cycle progression and the proliferation of neoplastic cells. Biochim Biophys Acta. 2002;1603(1):31–46. 12242109 10.1016/s0304-419x(02)00068-9

[12] Fast B, Kremp K, Boshart M, Steverding D. Iron-dependent regulation of transferrin receptor expression in *Trypanosoma brucei*. Biochem J. 1999;342 Pt 3:691–6. 10477281 PMC1220511

[13] Mussmann R, Engstler M, Gerrits H, Kieft R, Toaldo CB, Onderwater J, et al Factors affecting the level and localization of the transferrin receptor in *Trypanosoma brucei*. J Biol Chem. 2004;279(39):40690–8. 10.1074/jbc.M404697200 15263009

[14] Hirumi H, Hirumi K. Continuous cultivation of *Trypanosoma brucei* blood stream forms in a medium containing a low concentration of serum protein without feeder cell layers. J Parasitol. 1989;75(6):985–9. 2614608

[15] Dragset MS, Poce G, Alfonso S, Padilla-Benavides T, Ioerger TR, Kaneko T, et al A novel antimycobacterial compound acts as an intracellular iron chelator. Antimicrob Agents Chemother. 2015;59(4):2256–64. 10.1128/AAC.05114-14 25645825 PMC4356758

[16] Grintzalis K, Georgiou CD, Schneider YJ. An accurate and sensitive Coomassie Brilliant Blue G-250-based assay for protein determination. Anal Biochem. 2015;480:28–30. 10.1016/j.ab.2015.03.024 25837770

[17] He B, Zhang LL, Yue XY, Liang J, Jiang J, Gao XL, et al Optimization of Ultrasound-Assisted Extraction of phenolic compounds and anthocyanins from blueberry (Vaccinium ashei) wine pomace. Food Chem. 2016;204:70–6. 10.1016/j.foodchem.2016.02.094 26988477

[18] Maurya DK, Devasagayam TP. Antioxidant and prooxidant nature of hydroxycinnamic acid derivatives ferulic and caffeic acids. Food Chem Toxicol. 2010;48(12):3369–73. 10.1016/j.fct.2010.09.006 20837085

[19] Koide T, Nose M, Inoue M, Ogihara Y, Yabu Y, Ohta N. Trypanocidal effects of gallic acid and related compounds. Planta Med. 1998;64(1):27–30. 10.1055/s-2006-957360 9491765

[20] Andreo R, Regasini LO, Petronio MS, Chiari-Andreo BG, Tansini A, Silva DH, et al Toxicity and Loss of Mitochondrial Membrane Potential Induced by Alkyl Gallates in *Trypanosoma cruzi*. Int Sch Res Notices. 2015;2015:924670 10.1155/2015/924670 27347554 PMC4897139

[21] Breidbach T, Krauth-Siegel RL, Steverding D. Ribonucleotide reductase is regulated via the R2 subunit during the life cycle of *Trypanosoma brucei*. FEBS Lett. 2000;473(2):212–6. 10812077 10.1016/s0014-5793(00)01533-7

[22] Schnaufer A, Domingo GJ, Stuart K. Natural and induced dyskinetoplastic trypanosomatids: how to live without mitochondrial DNA. Int J Parasitol. 2002;32(9):1071–84. 12117490 10.1016/s0020-7519(02)00020-6

[23] da Silva Oliveira GL, de Freitas RM. Diminazene aceturate—An antiparasitic drug of antiquity: Advances in pharmacology & therapeutics. Pharmacol Res. 2015;102:138–57. 10.1016/j.phrs.2015.10.005 26470648

[24] Kuriakose S, Muleme HM, Onyilagha C, Singh R, Jia P, Uzonna JE. Diminazene aceturate (Berenil) modulates the host cellular and inflammatory responses to *Trypanosoma congolense* infection. PLoS One. 2012;7(11):e48696 10.1371/journal.pone.0048696 23144931 PMC3492428

[25] Zuma AA, Cavalcanti DP, Zogovich M, Machado AC, Mendes IC, Thiry M, et al Unveiling the effects of berenil, a DNA-binding drug, on *Trypanosoma cruzi*: implications for kDNA ultrastructure and replication. Parasitol Res. 2015;114(2):419–30. 10.1007/s00436-014-4199-8 25349143

[26] McKean PG. Coordination of cell cycle and cytokinesis in *Trypanosoma brucei*. Curr Opin Microbiol. 2003;6(6):600–7. 14662356 10.1016/j.mib.2003.10.010

[27] Gazitt Y, Reddy SV, Alcantara O, Yang J, Boldt DH. A new molecular role for iron in regulation of cell cycling and differentiation of HL-60 human leukemia cells: iron is required for transcription of p21(WAF1/CIP1) in cells induced by phorbol myristate acetate. J Cell Physiol. 2001;187(1):124–35. 10.1002/1097-4652(2001)9999:9999<::AID-JCP1061>3.0.CO;2-E 11241357

[28] Fu D, Richardson DR. Iron chelation and regulation of the cell cycle: 2 mechanisms of posttranscriptional regulation of the universal cyclin-dependent kinase inhibitor p21CIP1/WAF1 by iron depletion. Blood. 2007;110(2):752–61. 10.1182/blood-2007-03-076737 17429006

[29] Nairz M, Schroll A, Sonnweber T, Weiss G. The struggle for iron—a metal at the host-pathogen interface. Cell Microbiol. 2010;12(12):1691–702. 10.1111/j.1462-5822.2010.01529.x 20964797

[30] Huynh C, Andrews NW. Iron acquisition within host cells and the pathogenicity of Leishmania. Cell Microbiol. 2008;10(2):293–300. 10.1111/j.1462-5822.2007.01095.x 18070118 PMC2366998

